# Immunogenicity and Safety of a Newly Developed Live Attenuated Varicella Vaccine in Healthy Children: A Multi-National, Randomized, Double-Blinded, Active-Controlled, Phase 3 Study

**DOI:** 10.3390/vaccines11091416

**Published:** 2023-08-24

**Authors:** Ui Yoon Choi, Ki Hwan Kim, Hye-Kyung Cho, Dong Ho Kim, Sang Hyuk Ma, Young Youn Choi, Chun Soo Kim, Maria Rosario Capeding, Ilya Angelica Rochin Kobashi, Hun Kim, Ji Hwa Ryu, Su Jeen Lee, Ho Keun Park, Jong-Hyun Kim

**Affiliations:** 1 Department of Pediatrics, Eunpyeong St. Mary’s Hospital, College of Medicine, The Catholic University of Korea, Seoul 03312, Republic of Korea; uiyoon@catholic.ac.kr; 2 Department of Pediatrics, Incheon St. Mary’s Hospital, College of Medicine, The Catholic University of Korea, Seoul 21431, Republic of Korea; khkim99@catholic.ac.kr; 3 Department of Pediatrics, Gachon University College of Medicine, Incheon 21936, Republic of Korea; pdcho@ewha.ac.kr; 4 Department of Pediatrics, Korea Cancer Center Hospital, Seoul 01812, Republic of Korea; kdh@kirams.re.kr; 5 Department of Pediatrics, Changwon Fatima Hospital, Changwon 51394, Republic of Korea; pedma@naver.com; 6 Department of Pediatrics, Chonnam National University Medical School, Gwangju 61469, Republic of Korea; yychoi@jnu.ac.kr; 7 Department of Pediatrics, Keimyung University School of Medicine, Daegu 42601, Republic of Korea; cskim@dsmc.or.kr; 8 Department of Microbiology, Research Institute for Tropical Medicine, Manila 1781, Philippines; lerose@info.com.ph; 9 Centro de Investigacion Clinica del Pacifico, Acapulco 39680, Mexico; dr.rochin@cicpa.com.mx; 10 SK Bioscience, Seongnam 13494, Republic of Korea; ebolakim@sk.com (H.K.); jihwa.ryu@sk.com (J.H.R.); sujeen.lee@sk.com (S.J.L.); raulhead@sk.com (H.K.P.); 11 Department of Pediatrics, St. Vincent’s Hospital, College of Medicine, The Catholic University of Korea, Suwon 16247, Republic of Korea

**Keywords:** varicella, vaccine, immunogenicity, safety, children, clinical trial

## Abstract

Korean manufacturers have developed a new varicella vaccine, NBP608. This phase 3, randomized, double-blind, multicenter study aimed to compare the immunogenicity and safety of NBP608 in healthy children to those of Varivax^TM^ (control). Children aged 12 months to 12 years were randomized in a ratio of 1:1 to receive either NBP608 or the control vaccine. Serum samples were obtained before vaccination and within six to eight weeks after vaccination. In total, 499 participants (NBP608, n = 251; control, n = 248) were enrolled. The seroconversion rate (SCR) measured using a FAMA assay was 99.53% in the NBP608 group, and the lower limit of the 95% confidence interval (95% LCL) for the SCR difference (NBP608 minus the control) was 0.52%. This 95% LCL for the difference was higher than the specified non-inferiority margin of −15%. In an assessment using gpELISA, the SCR was 99.53% in the NBP608 group, and the 95% LCL for the SCR difference was 6.5%, which was higher than the specified non-inferiority margin of −15%. There were no significant differences between the NBP608 and control group with respect to the proportions of participants who demonstrated local and systemic solicited AEs. This study indicated that NBP608 had a clinically acceptable safety profile and was not immunologically inferior to Varivax^TM^.

## 1. Introduction

Varicella caused by varicella zoster virus (VZV) is a highly contagious disease that often occurs during childhood. Although the disease is often benign and self-limiting, varicella may cause serious complications, such as bacterial infection of skin and soft tissue, encephalitis, pneumonia, and sepsis. Varicella may be fatal in high-risk individuals, such as newborns, pregnant women, and those with congenital or acquired immunosuppression [1].

The implementation of varicella vaccination can lead to a significant reduction in varicella prevalence [2,3]. For countries where varicella is a salient public health issue, the WHO advocates the application of a single- or double-dose universal varicella vaccination (UVV) regimen [4]. Currently, about 40 countries have implemented UVV [5,6,7]; some countries have introduced vaccination for high-risk groups [8]. In some regions with high percentages of children, including China, India, and Southeast Asia, the varicella vaccine is available through the private sector [9,10].

The use of varicella vaccine is increasing worldwide [5]. Thus, a novel varicella vaccine, initially identified as NBP608, was developed by a Korean manufacturer [11]. The NBP608 vaccine was approved by the Ministry of Food and Drug Safety (MFDS) of Korea in 2018 and acquired pre-qualification certification from the WHO in 2019 [12]. Named SKYVaricella^TM^ for commercial production, the NBP608 vaccine is in use in Korea and is distributed to South American countries by the Pan-American Health Organization [13].

In this study, we report results of a phase 3 clinical trial conducted as a pre-licensure requirement of the MFDS of Korea. We aimed to evaluate the non-inferiority of immunogenicity and safety of NBP608 compared to those of Varivax^TM^ (Merck & Co., Inc., Kenilworth, NJ, USA).

## 2. Materials and Methods

### 2.1. Study Design and Participants

This phase 3, randomized, double blind, active controlled trial was conducted on 15 sites in Korea, 2 sites in the Philippines, and 2 sites in Mexico from July 2016 to June 2017. Eligible participants were healthy children aged 12 months to 12 years. In countries that recommend a single-dose varicella vaccination, it is administered at the age of 1 [5,6]; however, in particular cases, such as delayed vaccination or vaccination after exposure, older children may receive varicella vaccination. This study enrolled children between 12 months to 12 years of age and classified them into three age groups: 12–23 months, 24 months to 8 years, and 9 to 12 years. Investigators focused on enrolling children aged 1, in accordance with the recommended age of vaccination. The exclusion criteria were the receipt of a previous varicella vaccination, a history of varicella infection or exposure to VZV within the past four weeks, a body temperature of 38 °C or greater on the day of vaccination, hypersensitivity to gelatin or neomycin, a history of Guillain-Barre syndrome, congenital or acquired immunosuppression, the receipt of blood-derived products within five months of vaccination, the receipt of other investigational products within one month of vaccination or a plan to participate in another clinical trial during the study period, and ineligibility due to other reasons identified by the investigators.

This investigation adhered strictly to the principles stipulated by the International Conference on Harmonization Guidelines for Good Clinical Practice as well as the Declaration of Helsinki. The research protocol received approval from the Ministry of Food and Drug Safety (MFDS) of Korea; the governing drug authorities of both the Philippines and Mexico; and the Ethical Review Board of the participant site, namely St. Vincent’s Hospital of The Catholic University of Korea (Registration VC16BDGT0078). The trial was duly registered at Clinicaltrials.gov (NCT 03114943). In advance of trial enrollment, written consent was appropriately secured from the parents or legally appointed guardians of every participant.

### 2.2. Randomization and Masking

The participants were randomly assigned in a 1:1 ratio to receive either the investigational NBP608 or control vaccine. The Interactive Web Response System was used to randomly allocate participants, stratified by age, into the NBP608 or the control group. An unblinded pharmacist provided the vaccine in accordance with the randomization code, and an unblinded staff member administered the subcutaneous deltoid injection. Participants and their parents/legal representatives remained blinded to the allocations in the study and vaccine preparation processes.

### 2.3. Vaccines

The NBP608 investigational vaccine is a live attenuated varicella vaccine manufactured by SK Bioscience (Seongnam, Republic of Korea). The vaccine was derived from the Oka strain of VZV. The virus was propagated in MRC-5 human diploid cells and contained ≥ 2400 plaque forming units (PFUs) of lyophilized virus. Varivax^TM^ is also a live attenuated varicella vaccine derived from the Oka strain of VZV, was propagated in MRC-5 human diploid cells, and contained ≥ 1350 PFUs of lyophilized virus. Both vaccines were stored at 2–8 °C during the study period, and lyophilized pellets were reconstituted with 0.5 mL of sterile water immediately before injection.

Both NBP608 and Varivax^TM^ are based on the Oka strain, and each manufacturer has their own formulations. NBP608 was propagated in MRC-5 human diploid cells and attenuated sequentially in human embryonic cells, guinea pig embryonic cells, and human lung fibroblast cells. NBP608 is composed of raw materials, including a stabilizer such as hydrolyzed gelatin, and sucrose-phosphate-glutamate, and is lyophilized to stabilize the live virus [12]. The specific formulation and detailed manufacturing process are not publicized; therefore, we are limited in comparing the two vaccines. However, based on published information, the cell lines used in propagation and attenuation are similar between the two vaccines, but there are differences in the components and amounts of ingredients used as a stabilizer [12,14].

### 2.4. Immunogenicity Endpoints

The primary and secondary immunogenicity endpoints were established under the guidance of MFDS of Korea. The primary endpoint was assessed using a fluorescent-antibody-to-membrane-antigen (FAMA) assay, and the noninferiority of the investigational vaccine was confirmed if the lower limit of the 95% confidence interval (95% LCL) for the post-vaccination seroconversion rate (SCR) difference (that of the investigational vaccine minus that of the control vaccine) was greater than −15%. The secondary endpoints were measured for the SCR via a glycoprotein-based enzyme-linked immunosorbent assay (gpELISA) and the geometric mean titer (GMT) was assessed via FAMA and gpELISA. After the study’s completion, additional analysis was performed to test the noninferiority of the difference in SCRs (that of the investigational vaccine minus that of the control vaccine) measured via gpELISA, with a margin of −15%. Furthermore, post-vaccination GMT ratio analysis was conducted to assess whether or not the 95% LCL for the GMT ratio (the GMT of the investigational vaccine divided by that of control vaccine), as measured using both the FAMA assay and gpELISA, was equal to or greater than the non-inferiority margin of 0.5. Additionally, an exploratory noninferiority assessment of the SCR, as determined via both the FAMA assay and gpELISA, was conducted based on age groups. Age groups were categorized into three strata, 12–23 months, 24 months to 8 years, and 9 to 12 years, during the enrollment phase. The majority of participants belonged to the 12–23 month group, while the older age groups had a limited number of participants. For analysis purposes, the assessment was consolidated into two age categories: 12–23 months and 24 months–12 years.

### 2.5. Immunogenicity Assessment

The FAMA assay, the gold-standard method for detecting a neutralizing antibody, was performed according to a modified William’s method [15]. Blood samples were collected before vaccination and within six to eight weeks after vaccination. Sera were stored at −70 °C until analysis. To produce VZV-infected target cells with the cell-associated virus for use in the FAMA assay antigen, the MRC 5 cell line was cultured, inoculated with VZV, and harvested when cytopathic effects reached 50–70%. Two-fold serial dilutions of the sera were performed from 1:2 to 1:1024, and each diluted serum sample was mixed with the FAMA assay antigen. Fluorescein-isothiocyanate (FITC)-conjugated goat anti-human immunoglobulin G (IgG) was used as the secondary antibody. Two investigators examined the antigen-serum mixtures with a fluorescence microscope. Samples with a titer ≥1:4 were regarded as seropositive.

The measurement of antibody titers using gpELISA was performed with a Serion ELISA classic VZV IgG kit (Institut Virion/Serion GmbH, Wurzburg, Germany) in accordance with the manufacturer’s instructions. Consistent with the manufacturer’s recommendations, anti-VZV IgG concentrations >100 mIU/mL, 50–100 mIU/mL, or <50 mIU/mL were interpreted as protective, equivocal, or susceptible, respectively. As in previous studies, both equivocal and positive groups were considered seropositive [16]. The FAMA assay and gpELISA were performed at SK Bioscience Research Laboratory.

### 2.6. Safety Assessment

With respect to the safety endpoint, assessments were made of the proportions of participants within the NBP608 and control groups experiencing local solicited adverse events (AEs), systemic solicited AEs, and unsolicited AEs. Post-vaccination observations of the participants were conducted for a half-hour window, during which any local or systemic AEs or unsolicited AEs were duly documented by the study staff. Notably, local and systemic solicited AEs were reported by the participant’s parents or legal guardians spanning a seven-day period (Day 0 to Day 6), and a six-week observational window (Day 0 to Day 41) was allotted for the recording of unsolicited AEs. Aligning with the directives of the MFDS of Korea, the severity of local and systemic solicited and unsolicited AEs was categorized into mild (grade 1), moderate (grade 2), severe (grade 3), or potentially life-threatening (grade 4) [17].

Unsolicited AEs were delineated using terminology from the Medical Dictionary for Regulatory Activities version 19.1 [18]. Serious adverse events (SAEs), defined as potentially life-threatening events, those necessitating hospital admission, or those resulting in significant disability or death, were documented over a 26-week period.

Varicella-like rash was recorded for six weeks (Day 0 to Day 41). Samples from any newly appearing skin lesions were collected and underwent polymerase chain reaction (PCR) testing. PCR testing differentiated VZV from herpes simplex virus (HSV), another cause of blistering rash, and distinguished wild-type VZV from vaccine-derived VZV.

### 2.7. Statistical Analysis

Variations in categorical variables amongst the NBP608 and control groups were evaluated via a Chi square or Fisher’s exact test, where *p* values < 0.05 were deemed to have statistical significance. The Clopper–Pearson method was employed to compute the SCR and corresponding 95% CI for the immunogenicity assessment. Two-sided 95% CIs of the SCR differences were calculated using the Wald method. The GMT and corresponding 95% CI were calculated using an independent t-test on log-transformed antibody titers. The interval limits were re-transformed to the original scale. For safety assessment, the Clopper–Pearson method was applied to calculate the proportions of participants who experienced AEs and the corresponding 95% CI, and the Chi square or Fisher’s exact test was used for comparison. Based on previous data, the SCR in the NBP608 group was anticipated to be 76% or higher with a drop-out rate of approximately 30%. Therefore, a minimum of 244 participants in each vaccine group was required for a one-sided significance level of 0.025 and 90% power. All statistical analyses were conducted with SAS^®^ software, version 9.4 (SAS Institute, Cary, NC, USA).

## 3. Results

### 3.1. Study Participants

**Figure 1:**
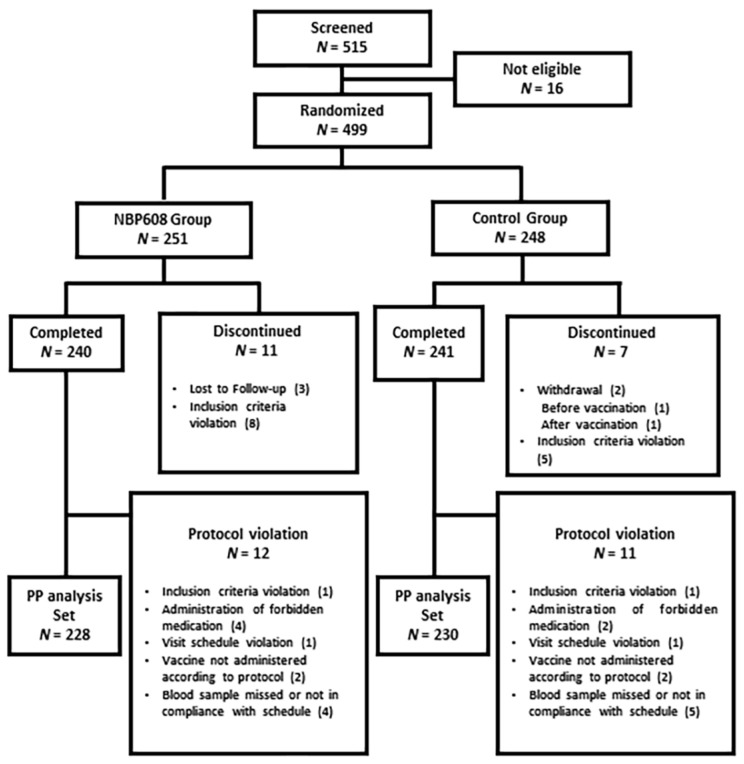
Flow diagram for selection of study participants. The number in parenthesis ( ) is the number of participants in corresponding disposition. Abbreviation: PP, per-protocol.

In total, 515 participants were screened, 499 eligible participants were randomly assigned to receive the NBP608 (n = 251) or control (n = 248) vaccine, and 498 who received vaccinations were included in a safety set. The flow of trial participants is shown in Figure 1. After excluding participants who failed to complete the study or who had violated the protocol, the per-protocol (PP) set included 228 participants in the NBP608 group and 230 in the control group. The demographic characteristics of the participants are presented in Table 1. Demographic characteristics of sex, age, weight, and height were comparable between the two groups.

### 3.2. Immunogenicity

The post-vaccination SCR measured via FAMA is presented in Table 2. The SCRs were 99.53% (95% CI: 97.40, 99.99) and 96.38% (95% CI: 92.99, 98.42) in the NBP608 and control groups, respectively. The difference in SCRs between the two groups (investigational group minus control group) was 3.15% (95% CI: 0.52, 5.78). The NBP608 group demonstrated non-inferiority to the control group, as the lower limit of the 95% CI of differences in the SCR was greater than −15%. Given the wide age range (12 months to 12 years) of participants, SCR was analyzed by age subset. In the subgroup aged 12–23 months, the difference in SCRs between the vaccine groups was 2.54% (95% CI: −0.08, 5.16), demonstrating NBP608 non-inferiority in this age group. In the 24 months to 12 years age group, the difference in SCRs between the two vaccine groups was 8.0% (95% CI: −2.63, 18.63). The NBP608 group demonstrated non-inferiority compared to the control group in this age group as well.

The SCR measured via gpELISA after vaccination is presented in Table 3. The SCRs were 99.53% (95% CI: 97.42, 99.99) and 88.79% (95% CI: 83.90, 92.61) in the NBP608 and control groups, respectively. The difference in SCRs between the two groups was 10.74% (95% CI: 6.50, 14.98). Therefore, the NBP608 group demonstrated non-inferiority to the control group. In the subgroup aged 12–23 months, the NBP608 group again demonstrated non-inferiority as the difference in SCRs between the vaccine groups was 10.61% (95% CI: 6.32, 14.89). In the 24 months to 12 years age group, the difference in SCRs between the two vaccine groups was 11.24% (95% CI: −5.78, 28.25), and NBP608 group’s non-inferiority in this age group was demonstrated.

**Figure 2:**
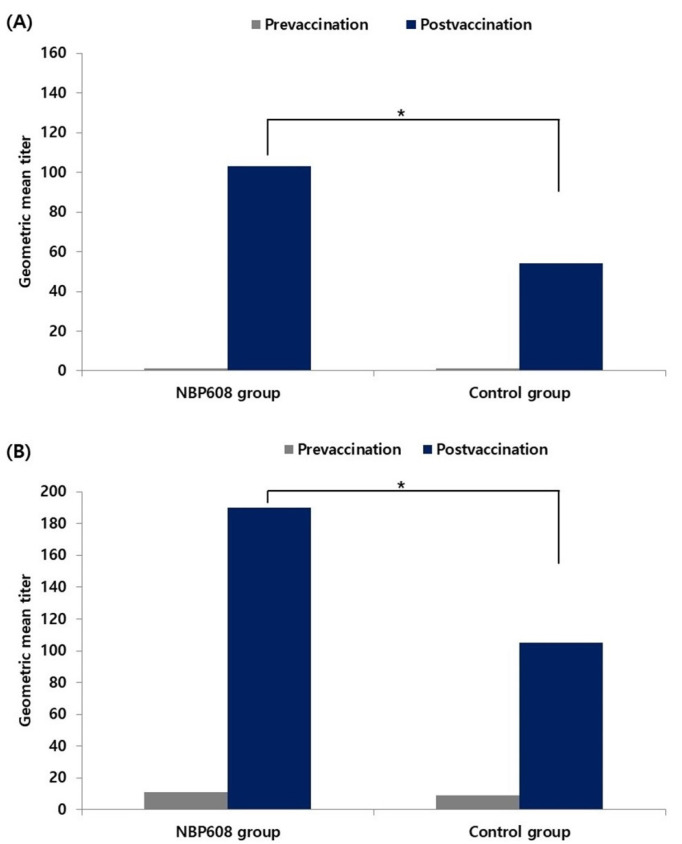
The pre- and post-vaccination geometric mean titers (GMT) for both the NBP608 and control groups. These were assessed using two different methods, (**A**) a fluorescent-antibody-to-membrane-antigen (FAMA) assay, where the post-vaccination GMT was found to be higher in the NBP608 group compared to that in the control group (* *p* < 0.001), and (**B**) a glycoprotein enzyme-linked immunosorbent assay (gpELISA), where similarly, the post-vaccination GMT was higher in the NBP608 group than that in the control group (* *p* < 0.001). The results include data from baseline seropositive participants.

Figure 2 presents pre- and post-vaccination GMTs regardless of the serostatus at the baseline, including that of baseline seropositive participants. As measured via the FAMA assay, GMT increased from 1.37 to 103.15 in the NBP608 group and from 1.22 to 54.22 in the control group. The post-vaccination GMT measured using the FAMA assay was higher in the NBP608 group (*p* < 0.001). As measured via gpELISA, the GMT increased from 10.88 to 189.77 in the NBP608 group and from 9.16 to 104.84 in the control group. The post-vaccination GMT measured using gpELISA was higher in the NBP608 group (*p* < 0.001). Table 4 presents the post-vaccination GMT ratio analysis; baseline seropositive participants were excluded. When assessed using the FAMA assay, the GMT ratio (the GMT of the investigational vaccine divided by that of the control vaccine) was 1.92 (95% CI: 1.55, 2.38). When assessed using gpELISA, the GMT ratio was 1.73 (95% CI: 1.53, 1.95). The 95% LCL of the GMT ratios, 1.55 as determined via the FAMA assay and 1.53 as determined via gpELISA, met the non-inferiority criterion of 0.5.

### 3.3. Safety

The safety set included 498 participants, and the reported AEs are presented in Table 5. No significant disparities were observed in the proportions of participants encountering local solicited AEs (*p* = 0.285), systemic solicited AEs (*p* = 0.756), or unsolicited AEs (*p* = 0.204) between the two groups, indicating analogous safety profiles. Erythema manifested as the most common local solicited AE, succeeded by pain/tenderness and swelling in both cohorts. Among the systemic solicited AEs, irritation was prevalent in both groups; infection and infestation were the most common unsolicited AEs. Most AEs were of grade 1 or 2, with no significant differences in the proportions of participants presenting grade 3 AEs between the groups (local solicited AEs, *p* = 0.075; systemic solicited AEs, *p* = 0.769; and unsolicited AEs, *p* = 0.686). None of the participants in either group demonstrated a grade 4 AE.

There were no significant differences in the proportions of participants who had varicella-like rashes (*p* = 0.106; 3.19% of NBP806 group versus 0.81% of control group). None of the participants contracted vaccine-derived VZV as determined by the PCR test. The proportions of participants with SAEs were not significantly different between the vaccine groups (*p* = 0.285). Eight participants, seven children with common childhood infectious diseases and one with a burn reported SAEs throughout the observation period. None of these were vaccine-related, and all eight children recovered.

## 4. Discussion

Within the context of this clinical trial, the immunogenicity and safety of a novel varicella vaccine were compared against those of Varivax™. Numerous methodologies for measuring antibodies against VZV have been established. The FAMA assay is considered the benchmark due to the high correlation of the FAMA titer with varicella protection [19,20]. Additionally, the gpELISA results demonstrated a strong correlation with the neutralizing antibody response [20,21,22]. Therefore, both the FAMA assay and gpELISA were selected for immunity testing in this study.

The manufacture of varicella vaccines is complex, and individual manufacturers have developed unique techniques and formulations with the goal of producing a vaccine that is devoid of VZV pathogenicity and capable of inducing a protective immune response [22,23,24]. Previously published pre-licensure studies have reported SCRs ranging from 85% to those greater than 90% [25,26,27,28,29]. The results for the NBP608 vaccine were consistent with those of previous studies and fulfilled the non-inferiority margin for immunogenicity in terms of the SCR measured using the FAMA assay and gpELISA. GMT ratio analysis suggested non-inferiority; the LCL of the 95% GMT ratio was higher than 0.5 in both the FAMA assay and gpELISA assay.

Varicella vaccines are generally safe [30,31,32,33], and no SAEs reported in this study were related to the investigational or control vaccines. Most of the AEs in both groups were grade 1 or 2. The proportions of participants for whom local and systemic solicited or unsolicited AEs were reported were comparable in the groups. In previous published studies on other varicella vaccines, varicella-like rash was reported in 4–6% of vaccine recipients [30,31]; in this study, varicella-like rash occurred in 3.19% of recipients of NBP608. Fever was reported to have occurred in 10−15% of varicella vaccine recipients, and most of these fever episodes were attributable to concurrent illness rather than vaccination [32,33]. In this study, fever related to vaccine was not clearly documented. In the NBP608 group, consistently with previous reports, 9.56% of participants reported fever. These results indicate the acceptable safety profile of the investigational vaccine.

There are limitations to this study that need to be addressed. First, immunogenicity was measured for only a short period, and additional studies evaluating long-term vaccine effectiveness and immune persistence are needed. Second, the study included a small number of children aged 24 months to 12 years, and future studies that include larger numbers of children in this age group are needed.

In conclusion, the NBP608 investigational vaccine satisfied the immunological non-inferiority and clinically acceptable safety profile criteria compared to those of the Varivax^TM^ control vaccine. The results will be informative to clinicians and healthcare authorities, particularly in regions that are currently in the process of implementing UVV or considering use of a varicella vaccine.

## Figures and Tables

**Table 1:** Demographic characteristics of the participants.

Characteristics	NBP608 Group (N = 251)	Control Group (N = 248)	*p*
Sex, No (%)			
Male	133 (52.99)	124 (50.0)	0.504
Female	118 (47.01)	124 (50.0)	
Age category, No (%)			
12–23 months	212 (84.46)	209 (84.27)	0.953
24 months–12 years	39 (15.54)	39 (15.73)	
Age, years			
Mean (SD)	2.40 (2.74)	2.43 (2.73)	0.904
Median (min, max)	1.42 (1, 11.92)	1.42 (1, 11.92)	
Body weight, kg			
Mean (SD)	12.34 (7.86)	12.15 (7.41)	0.781
Median (min, max)	9.90 (6.80, 55.20)	9.70 (7.10, 58.40)	
Height, cm			
Mean (SD)	84.17 (19.25)	83.82 (18.67)	0.833
Median (min, max)	77.6 (64.10, 153.50)	77.80 (64.30, 153.00)	

SD = standard deviation.

**Table 2:** Seroconversion rate and non-inferiority comparison between NBP608 and control groups using FAMA assay.

	NBP608 Group (N = 228)			Control Group (N = 230)			SCR Difference ^b^		
	n/M ^a^	%	(95% CI)	n/M ^a^	%	(95% CI)	Difference, %	(95% CI)	Non-Inferior ^c^
Overall	211/212	99.53	(97.40, 99.99)	213/221	96.38	(92.99, 98.42)	3.15	(0.52, 5.78)	Yes
Age group: 12–23 months	191/192	99.48	(97.13, 99.99)	190/196	96.94	(93.46, 98.87)	2.54	(−0.08, 5.16)	Yes
Age group: 24 months–12 yrs	20/20	100	(83.16, 100.0)	23/25	92.0	(73.97, 99.02)	8.0	(−2.63, 18.63)	Yes

FAMA = fluorescent antibody to membrane antigen, SCR = seroconversion rate, n = number of seropositive participants, M = number of participants for a given age group, CI = confidence interval; ^a^ FAMA assay. The number of baseline seronegative participants was 212 in the NBP608 group and 221 in the control group. The SCR was the proportion of participants who converted from being seronegative (FAMA titer < 1:4) before vaccination to seropositive (FAMA titer ≥ 1:4) after vaccination. ^b^ The difference is the NBP608 group’s SCR minus the control vaccine group’s SCR. ^c^ Non-inferiority was established when the lower limit of the 95% CI for the SCR difference was greater than −15%.

**Table 3:** Seroconversion rate and non-inferiority comparison between NBP608 and control groups using gpELISA.

	NBP608 Group (N = 228)			Control Group (N = 230)			SCR Difference ^b^		
	n/M ^a^	%	(95% CI)	n/M ^a^	%	(95% CI)	Difference, %	(95% CI)	Non-Inferior ^c^
Overall	213/214	99.53	(97.42, 99.99)	198/223	88.79	(83.90, 92.61)	10.74	(6.50, 14.98)	Yes
Age group: 12–23 months	193/193	100.0	(98.11, 100.0)	177/198	89.39	(84.25, 93.31)	10.61	(6.32, 14.89)	Yes
Age group: 24 months–12 yrs	20/21	95.24	(76.18, 99.88)	21/25	84.0	(63.92, 95.46)	11.24	(−5.78, 28.25)	Yes

gpELISA = glycoprotein enzyme-linked immunosorbent assay, SCR = seroconversion rate, n = number of seropositive participants, M = number of participants for a given age group, and CI = confidence interval; ^a^ gpELISA. The number of baseline seronegative participants was 214 in the NBP608 group and 223 in the control group. The SCR was the proportion of participants who converted from being seronegative (anti-VZV IgG <50 mIU/mL) before vaccination to seropositive (anti-VZV IgG ≥50 mIU/mL) after vaccination. ^b^ The difference is the SCR of the NBP608 group minus that of the control vaccine group. ^c^ Non-inferiority was established when the lower limit of the 95% CI for the SCR difference was greater than −15%.

**Table 4:** Antibody response comparison between NBP608 and control groups.

			NBP608 Group (N = 228)		Control Group (N = 230)		GMT Ratio ^a^	
	Time Point	M ^b^	GMT	(95% CI)	M ^b^ GMT	(95% CI)	Ratio	(95% CI)
FAMA	Pre-vaccination	212	1.08	(1.05, 1.11)	221, 1.08	(1.05, 1.11)		
	Post-vaccination	212	101.48	(87.97, 117.07)	221, 52.86	(45.02, 62.05)	1.92	(1.55, 2.38)
gpELISA	Pre-vaccination	214	8.12	(7.98, 8.26)	223, 8.12	(7.98, 8.25)		
	Post-vaccination	214	166.54	(153.03, 181.25)	223, 96.46	(88.64, 104.97)	1.73	(1.53, 1.95)

GMT = geometric mean titer, CI = confidence interval, FAMA = fluorescent antibody membrane antigen, and gpELISA = glycoprotein enzyme-linked immunosorbent assay. ^a^ Ratio is the GMT of the NBP608 group divided by that of the control vaccine group. ^b^ Only baseline seronegative participants were included in the GMT ratio calculation.

**Table 5:** Safety evaluation after vaccination.

	NBP608 Group (N = 251)			Control Group ^a^ (N = 247)			
	N	(%)	(95% CI)	N	(%)	(95% CI)	*p*
**Solicited local AE**	115	(45.82)	(39.54, 54.20)	125	(50.61)	(44.2, 57.00)	0.285
Grade 3	25	(9.96)	(6.55, 14.35)	14	(5.67)	(3.13, 9.33)	0.075
Pain/Tenderness	54	(21.51)		59	(23.89)		
Erythema	85	(33.86)		92	(37.25)		
Swelling	42	(16.73)		44	(17.81)		
**Solicited systemic AE**	64	(25.50)	(20.23, 31.36)	60	(24.29)	(19.08, 30.13)	0.756
Grade 3	7	(2.79)	(1.13, 5.66)	8	(3.24)	(1.41, 6.28)	0.769
Fever	24	(9.56)		22	(8.91)		
Irritation (whining)	39	(15.54)		33	(13.36)		
Sleepiness (feel drained)	27	(10.76)		20	(8.10)		
Headache	7	(2.79)		9	(3.64)		
**Unsolicited AE**	96	(38.25)	(32.21, 44.57)	81	(32.79)	(26.97, 39.03)	0.204
Grade 3	4	(1.59)	(0.44, 4.03)	2	(0.81)	(0.1, 2.89)	0.686
Infections and infestations	85	(33.86)		70	(28.34)		
Gastrointestinal disorder	6	(2.39)		6	(2.43)		
Respiratory disorder	3	(1.20)		3	(1.21)		
Varicella-like rash	8	(3.19)	(1.39, 6.18)	2	(0.81)	(0.10, 2.89)	0.106
Grade 4, any	0	(0)	-	0	(0)	-	
SAE	6	(2.39)	(0.88, 5.13)	2	(0.81)	(0.10, 2.89)	0.285

AE = adverse event, CI = confidence interval, SAE = serious adverse event. ^a^ A total of 248 participants were allocated to the control group, but 1 participant withdrew before vaccination. Therefore, the safety set of the control group included 247 participants.

## Data Availability

The datasets generated during and/or analyzed during the current study are available from the corresponding author upon reasonable request.
